# Supplementary material for: Growth of Renal Cancer Cell Lines Is Strongly Inhibited by Synergistic Activity of Low-Dosed Amygdalin and Sulforaphane
Source: Nutrients. 2024 Oct 31;16(21):3750. doi: 10.3390/nu16213750 (PMC11547972; doi:10.3390/nu16213750)
Supplement: Supplementary file 1 [file nutrients-16-03750-s001.zip › nutrients-3240494-supplementary.pdf]

Supplement S1

Vendor/Catalog No and RRID codes of the antibodies used.

Antibody	Vendor/Catalog No	RRID
Anti CDK1(clone 1)	BD Pharmingen 610037	AB_397454
anti-pCDK1/Cdc2 (clone 44)	BD Pharmingen 612306	AB_399621
anti-CDK2 (IgG2a, clone 55)	BD Pharmingen 610145	AB_397547
anti-Cyclin A (IgG1, clone 25)	BD Pharmingen 611268	AB_398797
anti-Cyclin B (IgG1, clone 18)	BD Pharmingen 610220	AB_397617
anti-PKBα/AKT (IgG1 clone 55)	BD Pharmingen 610861	AB_398180
anti-pAKT (clone 104A282)	BD Pharmingen 550747	AB_393864
anti-p19 (clone 52/p19 Skp1)	BD Pharmingen 610530	AB_397887
Anti-PTEN (Clone 26H9)	Cell Signaling 9556	AB_331153
anti-pCDK2 (Thr160)	Thermo Fisher PA5-104849	AB_2816322
anti-histone H3 (clone 3H1)	Cell Signaling 9717	AB_331222
anti-acetylated H3 (clone C5B11)	Cell Signaling 9649	AB_823528
anti-histone H4 (clone L64C1)	Cell Signaling 2960	AB_1147657
anti-acetylated H4 (polyclonal)	Millipore 07-328	AB_310524
Anti-Bax (B-9:sc-7480)	Santa Cruz sc-7480	AB_626729
anti-Bcl-2 (N-19:sc-492)	Santa Cruz sc-492	AB_2064290
anti-β-Actin (clone AC-15)	Sigma-Aldrich A1978	AB_476692

Supplement S2 A498

Western blots

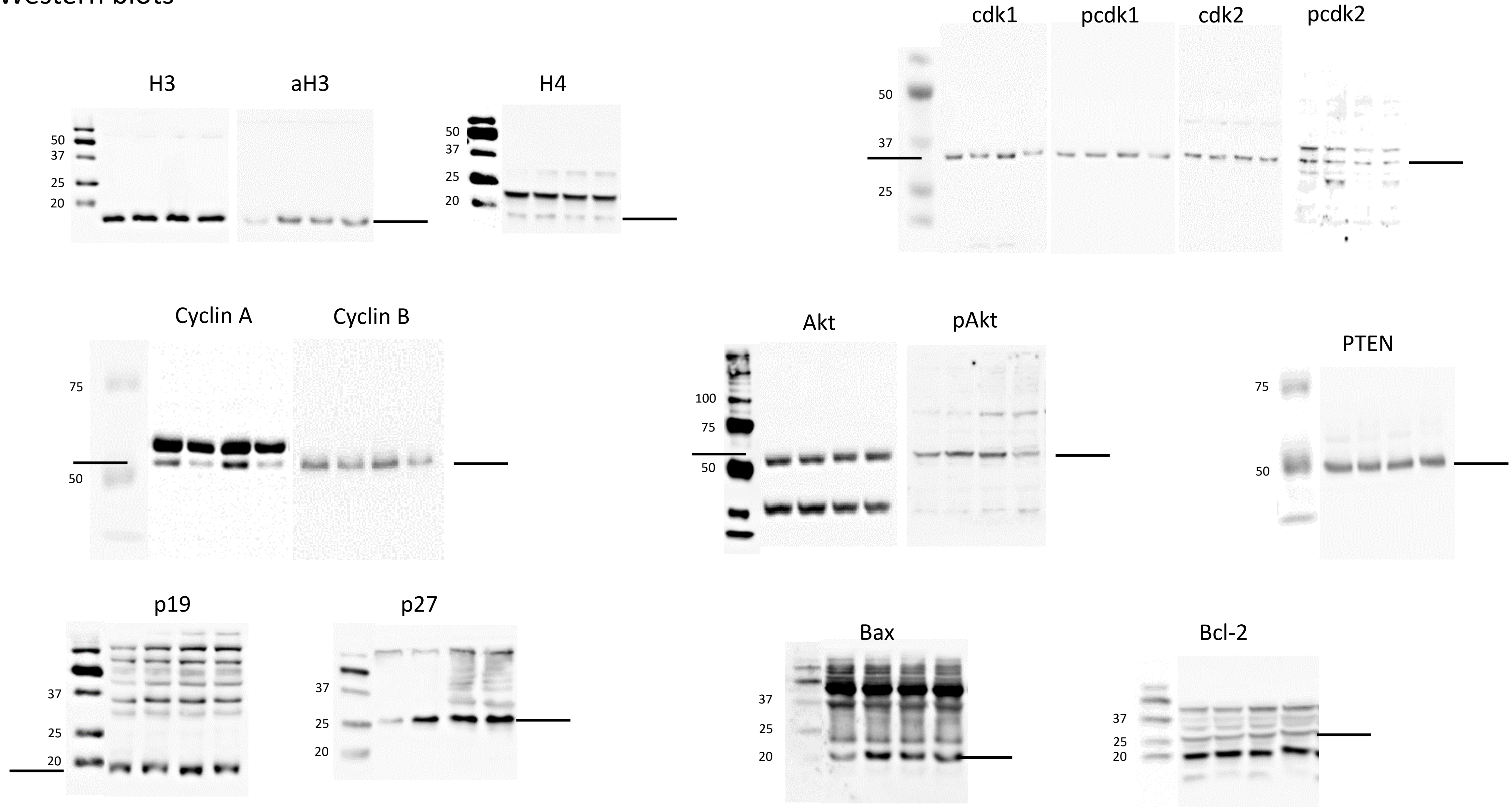

Caki-1

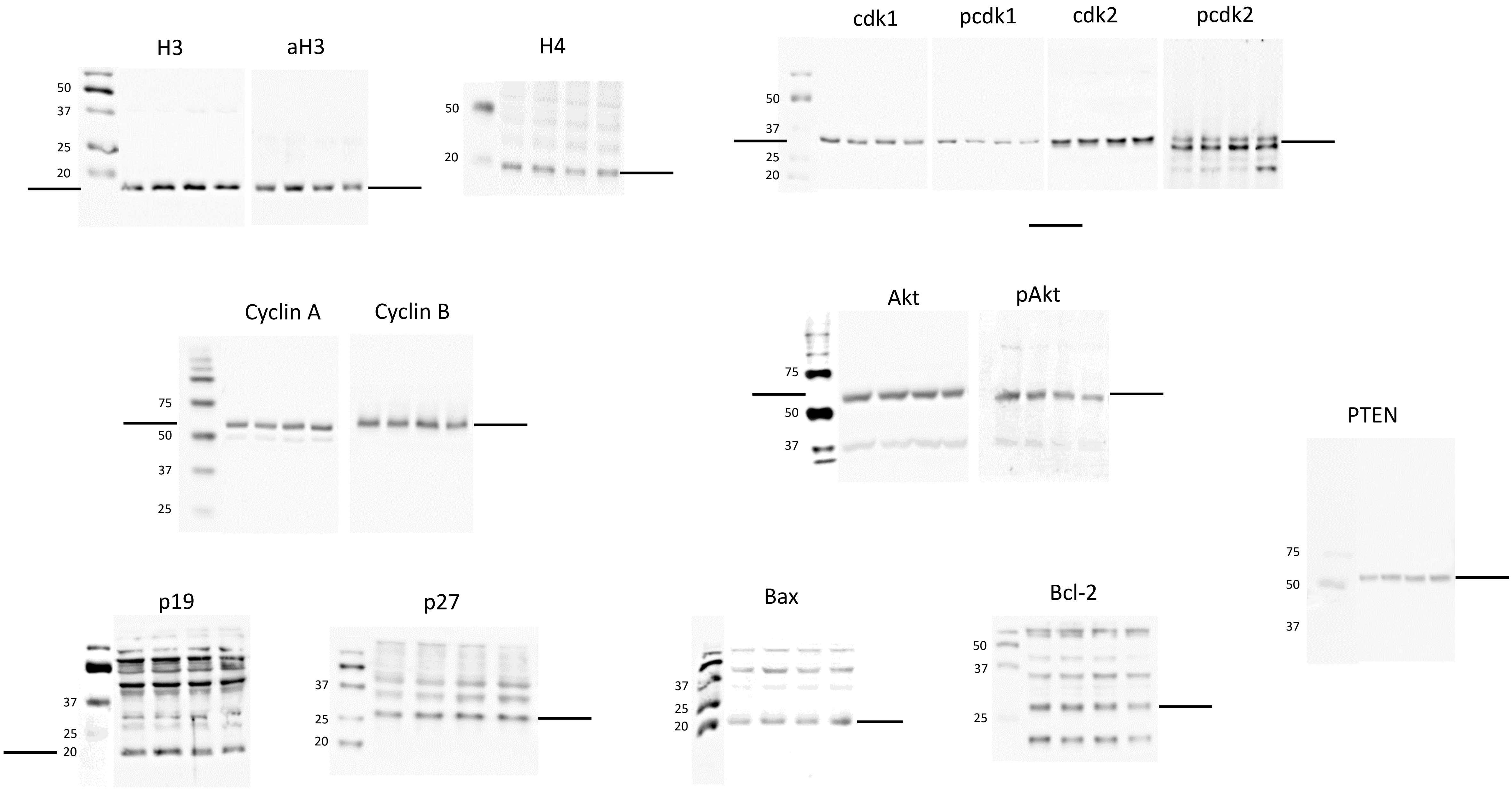
H3
aH3
H4
cdk1
pcdk1
cdk2
pcdk2
50
37
25
20
50
20
50
37
25
20
Cyclin A
Cyclin B
75
50
37
25
Akt
pAkt
75
50
37
PTEN
75
50
37
p19
37
25
20
p27
37
25
20
Bax
37
25
20
Bcl-2
50
37
25

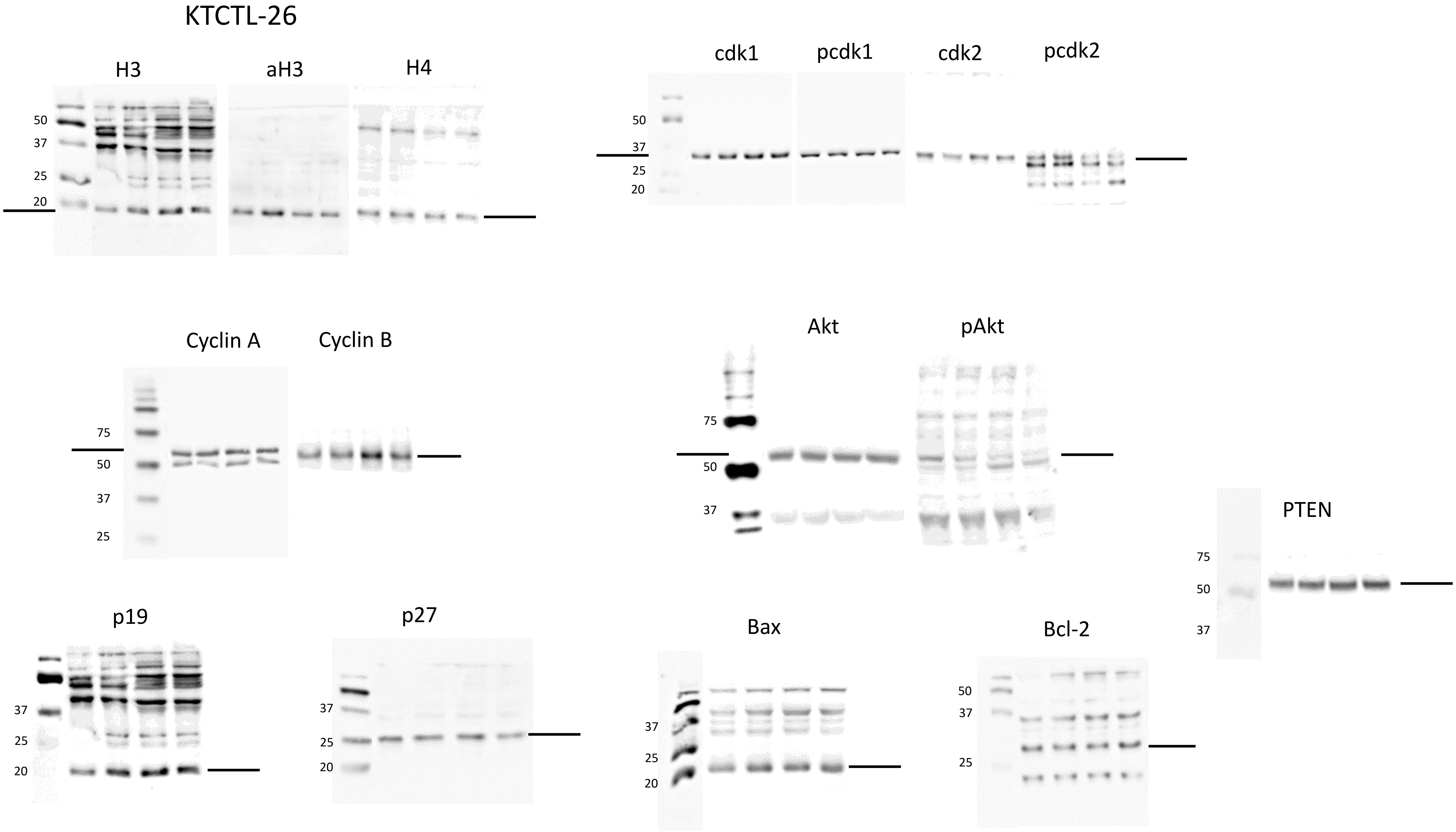
KTCTL-26
H3
aH3
H4
50
37
25
20
cdk1
pcdk1
cdk2
pcdk2
50
37
25
20
Cyclin A
Cyclin B
75
50
37
25
Akt
pAkt
75
50
37
PTEN
75
50
37
p19
37
25
20
p27
37
25
20
Bax
37
25
20
Bcl-2
50
37
25
